# The power of operon rearrangements for predicting functional associations

**DOI:** 10.1016/j.csbj.2015.06.002

**Published:** 2015-07-02

**Authors:** Gabriel Moreno-Hagelsieb

**Affiliations:** Department of Biology, Wilfrid Laurier University, 75 University Ave. W., Waterloo, ON N2L 3C5, Canada

**Keywords:** Operons, Operon rearrangement, Operon predictions, Functional associations, Genomic context, Conservation of gene order, Comparative genomics

## Abstract

In this mini-review I aim to make the case that operons might be the most powerful source for predicted associations among gene products. Such associations can help identify potential processes where the products of unannotated genes might play a role. The power of the operon for providing insight into functional associations stems from four features: (1) on average, around 60% of the genes in prokaryotes are associated into operons; (2) the functional associations between genes in operons tend to be highly conserved; (3) operons can be predicted with high accuracy by conservation of gene order and by the distances between adjacent genes in the same DNA strand; and (4) operons frequently reorganize, providing further insight into functional associations that would not be evident without these reorganization events.

## 1 Introduction

Operons, were first defined as a set of genes transcribed from an operator [1]. By extension, here I define them as two or more adjacent genes in the same strand transcribed into a single messenger RNA (a polycistronic mRNA). It is somewhat expected, as it has been corroborated [2,3], that most genes transcribed into a polycistronic mRNA should code for products that work together. Given the traditionally perceived importance of operons in co-regulating genes whose products functionally interact, they have been central in the field of comparative genomics aiming at predicting functional associations. In this mini-review, I attempt at further justifying this focus. I also attempt at providing evidence that predicted operons in one organism can give clues to functional associations in another organism. Because of the potential transference of functional associations from operons in one organism into genes found in another organism, the power of predicted operons for providing potential associations expands exponentially.

This review is not intended to be a comprehensive view on the methods for predicting functional associations, nor is it intended as a comprehensive view at methods for predicting operons. For further learning about predicting functional associations by genomic context, and derived methods, the reader can consult such works as [4–8]. For methods on operon predictions the reader can check [9–12] among others.

## 2 Genes without functions and the panorama of potential interactions

Since the very first genome sequences became available, researchers noticed that a large amount of genes could not be functionally annotated by looking for homologues (see for example [13]). Case in point, a third of the genes in the model organism *Escherichia coli* K-12 MG1655 remain functionally uncharacterized [14] (this is still true today). Inspired by this fact, scientists started proposing methods for predicting operons by methods other than those based in direct homology (for example [15] and references therein).

Predicting functions by methods other than direct homology involves the finding of interactions with the expectation that interactions between unannotated genes and genes with characterized functions (or functionally-annotatable by direct homology), would help predict the functions of the uncharacterized genes. The idea behind transference of functions has been aptly called “guilt by association” [16]. Three main ideas for predicting functions by association appeared: (i) phyletic patterns or phylogenetic profiles [17,18], based on the expectation that if the products of two genes functionally interact, then the genes should co-occur, since the product of one gene would be expected to be useless without the product of the other; (ii) conservation of adjacency [19,20,21], where genes remaining next to each other across genomes are expected to functionally interact; and (iii) gene fusions [22,21], where, if two separate genes in one genome appear as a single fused gene, they might functionally interact.

To put the above ideas in perspective, it is useful to think of the problem of predicting functional interactions as the problem of finding actual interacting pairs among the maximum number of pairs available for exploration in a genome. This exploratory space (*E*) can be calculated from the total number of annotated genes (*N*) as:

(1) $$E=\frac{N\left(N-1\right)}{2}\;\text{.}$$

Let us consider the case of *E. coli* K12 MG1655 as an illustration. The version of the genome available by November 2014 contains 4138 coding genes. This translates into an exploratory space of 8,559,453 pairs. Considering that the genome consists of a circular chromosome, the maximum number of pairs that could be explored by conservation of gene order would be 4138 (the same as the number of genes), less than 5% of the exploratory space. In theory, the exploratory potential would be much larger for gene fusions, since genes do not have to be adjacent in a genome of interest in order to find them fused in another genome. However, in practice we have found few fused genes (Fig. 4B). The potential for phylogenetic profiles would appear to be the largest. After all, there is no need for the genes to be adjacent in any of the genomes analyzed. However, co-occurrence analyses seem to produce few high-quality annotations (Fig. 4B), perhaps precisely because the background is the total exploratory space, which might consist of a large fraction of true negatives. Thus the question becomes: is it possible to expand on high-quality functional interactions and avoid the enormous number of potential negatives in the exploratory space? The answer seems to be the analyses of operon rearrangements.

## 3 Operons can be predicted

**Fig. 1:**
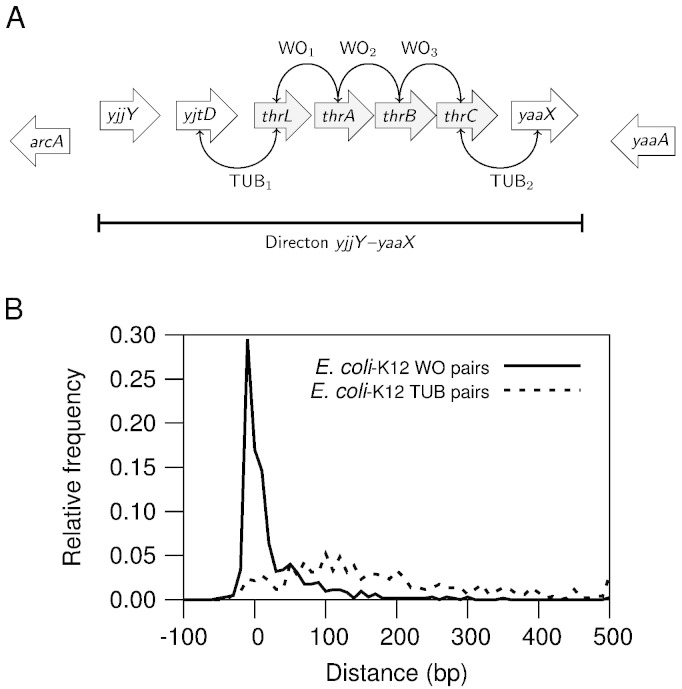
Intergenic distances. (A) Representation of a directon, a stretch of adjacent genes in the same strand with no intervening gene in the opposite strand. The figure shows an operon within the directon, pairs of genes in operons (WO) and transcription unit boundaries (TUB). (B) The distances between genes in operons tend to be short compared to those between genes in different transcription units. The distances were binned at ten base intervals to calculate relative frequencies.

The problem of predicting operons could be conceptualized as the problem of finding transcription unit (TU) boundaries within a stretch of adjacent genes in the same strand with no intervening genes in the opposite strand. We call these stretches of genes in the same strand “directons” [2] (Fig. 1A).

### 3.1 Predicting operons by intergenic distances

An initial assumption about genes in operons was that, since there is no need for signals between co-transcribed genes, the distances between genes in the same operon would be shorter than those between genes in different TUs (Fig. 1). The assumption was first confirmed using known operons gathered from the literature as found in RegulonDB [23], mapped into the genome of *Escherichia coli* K12 to find boundaries between TUs [2]. The finding was key in the success of operon predictions from the first time it was used [2,24]. Intergenic distance continues to be the most informative feature for operon predictions [25–27,12].

### 3.2 Predicting operons by conservation of gene order

Another initial assumption was that operons would have a tendency to be conserved across prokaryotic organisms. Accordingly, some early results in comparative genomics found that adjacent genes in the same strand tend to be better conserved next to each other across genomes than adjacent genes in opposite strands [19,28]. Furthermore, the comparison of conservation of genes in the same strand against that of genes in different strands allowed for high-confidence prediction of operons in genomes with no experimental information on TU organization [29], and for the confirmation that genes in operons have the same tendencies for short intergenic distances among prokaryotes as that observed in *Escherichia coli* [30,24,31].

## 4 Most genes in prokaryotes are in operons

**Fig. 2:**
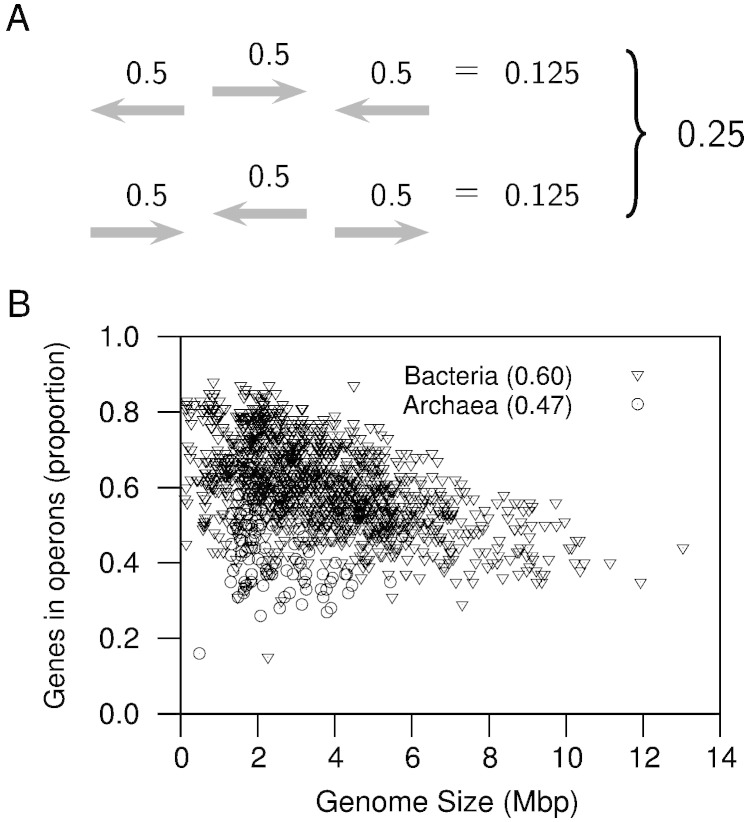
Proportion of genes in operons. (A) The number of genes surrounded by genes in the opposite direction should be approximately equal to 25% of the genes in single-gene transcription units (TUs). Thus, the proportion of genes in operons, TUs with more than one gene, can be estimated from the difference between the total number of genes and those in single-gene TUs. (B) There is variation in the proportion of genes in operons across genomes. The figure shows calculations for a non-redundant collection of complete genomes from NCBI's RefSeq [33] available by November 2014. Overall, the proportion averages 60% overall (60% in Bacteria, and 47% in Archaea).

Some years ago, Cherry [32] published operon estimates based on very simple assumptions. For example, if TUs can be found on either DNA strand, then approximately one fourth of all TUs should be in a strand by themselves. That is, their neighboring TUs would be found in the opposite strand (Fig. 2A). Since there is no reason to expect the length of the TU to influence which ones would be found in a directon by themselves, it follows that one fourth of the subset of TUs producing monocistronic RNAs should be found surrounded by TUs in the opposite strand. These single-gene TUs would be evident as single-gene directons (singletons). Thus, the proportion of genes transcribed into monocistronic RNAs should be approximately equal to the number of singletons multiplied by four. If we then wanted to know the number of genes in operons, we would only have to subtract this number from the total number of genes (*T_genes_*). Thus, the proportion of genes in operons would be calculated as:

(2) $$P_{opn}=\frac{T_{\mathrm{genes}}-\left(4\times N_{\mathrm{singletons}}\right)}{T_{\mathrm{genes}}}\text{.}$$

Of course, the formula assumes that the only reason why there would be a tendency for adjacent genes to remain in the same directon is if they are in operons. Such might not be the case. For example, a tendency towards staying in the leading strand has been observed for genes close to origins of replication. However, careful analyses of operons in *Escherichia coli* K12, has shown that, if operons are not the only reason for adjacent genes to remain in the same strand, then they might be the main reason, with no noticeable influence from other factors at the genomic scale [31].

Using the formula above, I have continued to calculate the proportion of genes in operons as the database of prokaryotic genomes has grown [31] (https://microbiome.wordpress.com/research/operon-estimates/). For this mini-review, I used the complete prokaryotic genomes available at NCBI's RefSeq [33] by November 2014. I kept 1408 non-redundant genomes by clustering the original 2765 using DNA tetra-nucleotide signature distances [34]. The cutoff threshold was a distance of 0.04, which roughly corresponds to a species level [34]. Since the first calculation [31], the average proportion of genes in operons across prokaryotes has remained at around 0.60 (Fig. 2B). Therefore, most genes in prokaryotes might be associated into operons. Operons might be the most common way in which genes whose products functionally interact are transcribed together.

## 5 Operons display highly conserved functional associations

**Fig. 3:**
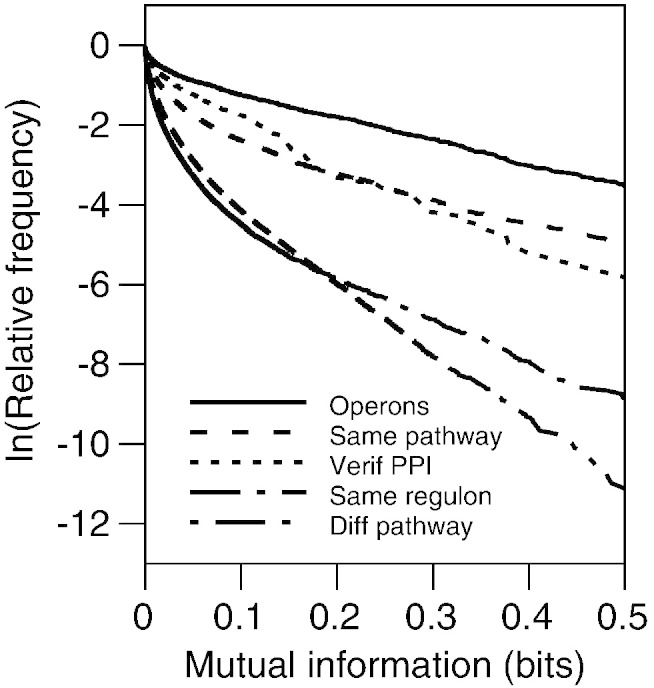
Comparing evolutionary conservation. Mutual information measures the codependence of two variables. Higher values indicate stronger codependence, which makes mutual information useful for measuring gene co-occurrence across genomes [3,46]. To compare the co-occurrence of pairs of genes with different kinds of functional interactions, the figure shows the proportion of gene pairs left in each category as the mutual information threshold increases. Genes in operons have a higher tendency to co-occur across genomes than genes associated in other ways. The higher co-occurrence can be interpreted as a higher tendency towards conservation of the implied functional interaction.

A comparison of the conservation of experimentally-known functional associations of *Escherichia coli* K12 has found that genes in operons tend to have the most evolutionary stable functional associations [3]. Evolutionary conservation was measured as the tendency of associated genes to co-occur across prokaryotic genomes. The method is called p-cubic, because it consists of the comparison of curves derived from the mutual information of phylogenetic profiles, in other words, *p*rofiles of *p*hylogenetic *p*rofiles (p-cubic). Essentially, the tendency for a group of gene pairs to co-occur contrasts with the lack of such tendency in another group, because the curve of the former runs above the curve in the latter (Fig. 3). This is very similar to curves used in previous studies [35].

The experimentally-determined functional modules compared were pairs of genes in the same operon, genes coding for products working in the same biochemical pathway, genes coding for proteins that physically interact, and genes associated via proteins that regulate transcription [3]. The work found that genes in the same operon had the p-cubic curve showing the highest tendency for co-occurrence. This result holds with current datasets (Fig. 3).

It is therefore tempting to conclude that operons might reveal functional associations that tend to be conserved across prokaryotes.

## 6 Rearranged operons: a large window into functional associations

**Fig. 4:**
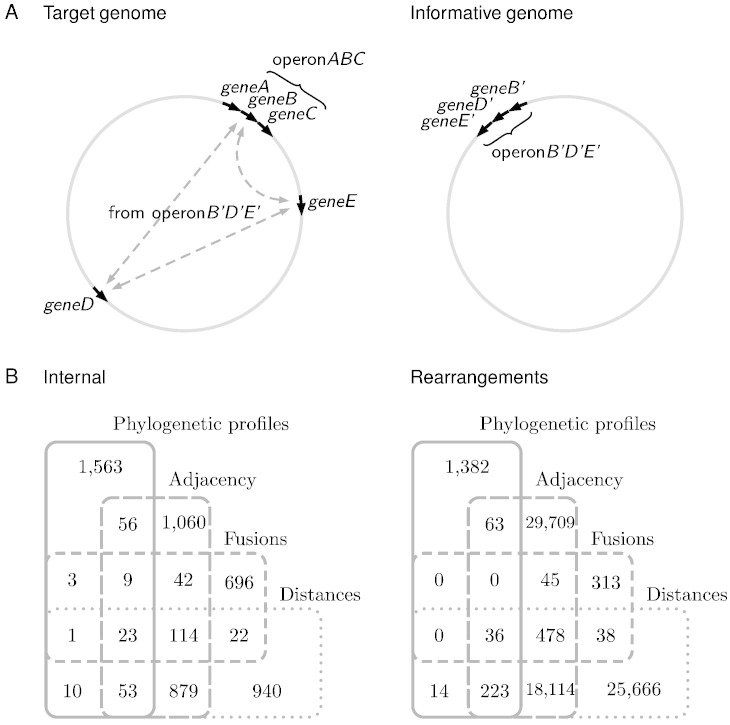
Predictions and operon rearrangements. (A) Operons in informative genomes help predict interactions between separated genes in a target genome. The dotted arrows between *geneB*, *geneD* and *geneE* show predicted interactions in the target genome. These interactions were transferred by orthology from the predicted operon *B*′*D*′*E*′ in the informative genome to the corresponding genes in the target genome. (B) The number of predicted interactions increases substantially when predictions based on operons in informative genomes (rearrangements) are added to those based on operons in the target genome alone (internal).

Early in comparative and functional genomics, Galperin and Koonin [36] suggested that, if operons frequently rearranged, then predicting operons could potentially be a powerful source for predicted functional associations. They pointed out that no successful method existed yet for predicting operons. Close to that time, a successful method for predicting operons appeared in the literature [2]. The idea for expanding predictions beyond those produced within a single genome works as follows (Fig. 4A): genes separated in a genome of interest (or target genome), could be inferred to functionally interact if their orthologs were found to be in the same operon in some other genomes (the informative genomes). This idea has been implemented on the basis of operons predicted by conservation of gene order [37–39], and was later expanded to include operons predicted by intergenic distances [40].

It is to be expected that operon rearrangements increase the number of available predicted functional associations. Actually, the number of predictions increase several fold (Fig. 4B). Putting together all the information presented in this mini-review, if operons represent the most evolutionarily stable functional associations, and if they can be predicted with high accuracy, and if they rearrange in a functionally-meaningful way, then operons are a very powerful source of information for predicting functional associations in prokaryotes.

## 7 Caveats and future directions

As mentioned above, methods for predicting functional associations based on operon rearrangements have been successful in assigning functions to previously uncharacterized genes [14]. The quality of predictions has also been demonstrated [40,14,41,42]. However, as more genomes are sequenced, there is a danger that false positives might be enough in number to lower the quality of overall predictions. Since genome rearrangements are frequent, the potential for non-interacting genes to appear adjacent and have intergenic distances proper of operons in at least some genomes increases. Some solutions to the problem might be provided by using the structure of the predicted network of interactions. For example, by making sure that connected genes share most other connections to other genes [14,41,42].

It would also be advisable to investigate further methods for predicting operons. For example, the intergenic distance method mostly presented here has a maximum accuracy of around 0.82 correct predictions as evaluated in both *Escherichia coli* and *Bacillus subtilis* [24]. Other methods claim accuracies above 0.90 [10]. Such methods should be further evaluated and explored so as to improve predictions and better access the power of rearranged operons for predicting functional associations. Improved operon predictions across prokaryotes will be highly dependent on the development of databases containing high-throughput operon mappings across organisms, such as those derived from RNA-seq analyses present in the DOOR database [43].

Another problem is that genome annotations might contain several false genes, which might artificially interrupt a director, and thus break an operon. Related to this point, some operons have been reported to contain genes in opposite strands (for example: [44,45]). Both these problems, however, might be compensated by the presence of similar operons in other genomes that do not contain the interrupting gene.

Other problems with genome annotations is the potential for mistaken start codons. The first predictions based on intergenic distances in *Escherichia coli* did not produce positive predictive values, proportion of true positives in predicted operon gene pairs, above 0.86. After genome resequencing and reannotations, the method has produced positive predictive values above 0.90 (author's unpublished observation).

Further complications arise from the presence of nested and overlapping TUs. These constitute around 10% of the TUs reported in RegulonDB [23], and around 20% of those reported in DBTBS [45]. The distances between genes in nested TUs tend to be at the zone where predictions are less confident. Again, it is possible that less complex operons might exist in other genomes and thus compensate for this problem.

Overall, the case for operons as a powerful source for predicting functional associations seems to be well founded. However, some considerations, like those listed above, still make it a field in need for further development, development that seems to be worth pursuing.
